# Clinical Utility of Molecular Tests for Guiding Therapeutic Decisions in Bloodstream Staphylococcal Infections: A Meta-Analysis

**DOI:** 10.3389/fped.2021.713447

**Published:** 2021-08-05

**Authors:** Ke Chen, Aijaz Ahmad Malik, Yun-Jian Sheng, Sarfraz Ahmed, Changfeng Sun, Cun-Liang Deng, Suvash Chandra Ojha

**Affiliations:** ^1^Department of Infectious Diseases, The Affiliated Hospital of Southwest Medical University, Luzhou, China; ^2^Southwest Medical University, Luzhou, China; ^3^Faculty of Medical Technology, Center of Data Mining and Biomedical Informatics, Mahidol University, Bangkok, Thailand; ^4^Department of Basic Sciences, University of Veterinary and Animal Sciences Lahore, Narowal, Pakistan

**Keywords:** bloodstream infection, NAAT accuracy, staphylococcal infections, pediatric population, meta-analysis

## Abstract

**Background:** Treatment of bloodstream staphylococcal infections (BSI) necessitates the prompt initiation of appropriate antimicrobial agents and the rapid de-escalation of excessive broad-spectrum coverage to reduce the risk of mortality. We, therefore, aimed to demonstrate the diagnostic accuracy of nucleic acid amplification tests (NAAT) for the identification of methicillin-resistant *S. aureus* (MRSA) and methicillin-sensitive *S. aureus* (MSSA) in clinically suspected patients.

**Methods:** Until November 23, 2020, databases including PubMed, Scopus, Embase, and Web of Science were scanned for eligible studies. A bivariate random-effects model was used for meta-analysis of the 33 included studies obtained from 1606 citations, and pooled summary estimates with 95% confidence intervals (CI) were generated.

**Results:** Twenty-three studies (*n* = 8,547) assessed NAAT accuracy for MSSA detection, while three studies (*n* = 479) evaluated MRSA detection in adults. The pooled NAAT sensitivity and specificity for MRSA in adults was higher [sensitivity: 0.83 (95% CI 0.59–0.96), specificity: 0.99 (95% CI 0.98–1.0)] as compared to MSSA [sensitivity: 0.76 (95% CI 0.69–0.82), specificity: 0.98 (95% CI 0.98–0.99)]. Similarly, eight studies (*n* = 4,089) investigating MSSA in pediatric population reported higher NAAT accuracy [sensitivity: 0.89 (95% CI 0.76–0.96), specificity: 0.98 (95% CI 0.97–0.98)] compared to adults. Among NAA tests, Septi*F*ast (real-time PCR, commercial) was frequently applied, and its diagnostic accuracy corresponded well to the overall summary estimates. A meta-regression and subgroup analysis of study design, sample condition, and patient selection method could not explain the heterogeneity (*P* > 0.05) in the diagnostic efficiency.

**Conclusions:** NAAT could be applied as the preferred initial tests for timely diagnosis and BSI management.

## Introduction

Bloodstream staphylococcal infection (BSI) is an urgent medical issue due to its rising incidence, associated poor outcome and the emergence of high rates of secondary infections such as osteomyelitis, septic arthritis, infective endocarditis and septic metastases (1, 2). Many surveillances worldwide recorded a rise in the incidence of BSI to varying degrees, including Thailand (27.4%) (3), France (24%) (4) and Brazil (40–70%) (5), but the majority of documented cases are in adults. Pediatric incidence statistics are minimal, but infants younger than 1 year have been reported as having a higher incidence compared to adolescents (6). Especially in developed countries, methicillin-resistant *S. aureus* (MRSA) has been observed in over 60% of all isolated *S. aureus*, and associated MRSA mortality rates have reportedly been higher than methicillin-susceptible *S. aureus* (MSSA) (7). The burden of BSI is utterly immense in both clinical (8) and economic terms (9), with mortality rates ranging between 20 and 70% (10, 11). Thus, rapid identification of the causative agents and detection of resistance markers in patients with BSI, such as the *mecA* gene, may provide clinically vital information to guide effective care on time, leading to improved patient outcomes.

Vancomycin, a glycopeptide antimicrobial agent, has frequently been included as empirical therapy for suspected BSI (12), along with the awareness that delays in initiating effective antimicrobial therapy may affect patient outcomes. Once identification and susceptibility of microorganisms are established, treatment can be optimized to target isolated bacteria, including discontinuation of vancomycin when MSSA is present. Though safe, this approach exposes the patient to wide-spectrum antibiotic overuse. Even short anti-MRSA treatment courses may alter host flora, expose to drug-induced toxicity, escalate multidrug-resistant pathogens, treatment-related side effects, and increase hospitalization costs (13). Vancomycin, the preferred antibiotic solution for MRSA infections, is also less effective than oxacillin in the treatment of MSSA infections (14). If the initial antibiotics are inadequate and changed after the diagnostic tests are available, the mortality rate does not improve significantly. Therefore, it is increasingly important to balance these two competing interests, namely the need for complete coverage while avoiding unnecessary medications.

Blood cultures currently represent the primary method for determining the etiology of the BSI (15). This traditional culture-based approach can be time-consuming, including growth-based assays, colony morphology and microdilution resistance tests. It takes about 48–72 h to classify the causative organism, even with a positive blood culture. A positive microbiological diagnosis in BSI can only be made in ~20–30% of cases with a substantially higher false-negative rate (16, 17), and requires a certain amount of sample to begin the cultivation process. Nevertheless, obtaining adequate amounts of blood from neonates for culture is often difficult (18). Furthermore, samples collected after antibiotic exposure may reduce culture-based bacterial detection. As a result, the patients will likely miss the optimal chance of treatment. Therefore, if no pathogenic bacterial agent is detected, sepsis diagnosis is based solely on clinical symptoms, often coupled with an increase in essential biomarkers such as C-reactive protein or procalcitonin (19). For these reasons, there is an obvious need for a more rapid, yet precise tool that uses a limited blood volume to detect organism and guide antibiotic choices in patients with suspected BSI.

While the pathogen culture remains the gold standard, molecular amplification tests, which typically have a shorter turnaround time, can drastically reduce the critical time to initiate preventive and therapeutic strategies, including appropriate antibacterial therapy. Unlike traditional blood culture, NAAT relies on detecting bacterial DNA rather than the recovery of viable bacteria and is less affected by antibiotic pre-administration. A previous study demonstrated a decrease in the length of anti-MRSA antibiotics in patients with BSI following a rapid diagnostic test (20). Several studies have assessed the importance of molecular techniques including PCR, real-time PCR, GeneXpert, LAMP, and FilmArray (21–25), but literature on the relevance of these tests to timely BSI management is widely scattered for any meaningful interpretation. Given the need to make clinical decisions among the pediatric and adult population, we have systematically reviewed and analyzed the available data to demonstrate NAAT diagnostic accuracy compared with microbiological culture.

## Methods

### Search Strategy

This systemic review was carried out in compliance with the guidelines for Preferred Reporting Items for Systematic Reviews and Meta-Analyses (PRISMA) (26). A computerized search of the relevant studies without any restrictions was performed through PubMed (available since April 01, 1991), Scopus (available since April 01, 1992), Embase (available since July 01, 1992), Web of Science (available since December 01, 1999), and a reference study of the retrieved papers published until November 23, 2020. The search included a variation of the Boolean “OR” and “AND” operators with the following medical subject headings (MeSH): “*Staphylococcus aureus*,” “*S. aureus*,” “methicillin-resistant *S. aureus*,” “MRSA,” “Bloodstream infection,” “Blood infection,” “Blood culture,” “Bacteremia,” “Septicemia,” “Sepsis,” “Blood poisoning,” “Nucleic acid amplification,” “NAAT,” “Molecular assay,” “Loop-mediated isothermal amplification,” “LAMP,” “Polymerase chain reaction,” “PCR,” “Ligase chain reaction,” “LCR,” “Real-time PCR,” “qPCR,” “RT-PCR,” “Amplicor,” “Septi*F*ast,” “ProbeTec,” “Roche,” “Gen-Probe,” “FilmArray,” “Cepheid,” Abbott,” “Sensitivity,” “Specificity,” and “Accuracy.” No effort was made to obtain data from unpublished studies.

### Study Selection

The MeSH terms were used to search through electronic databases for all relevant citations, and duplicates were carefully removed using the EndNote X9 software (Thomson Reuters, New York, NY, USA). The records obtained were initially scrutinized by reviewing titles and abstracts, and subsequent analysis excluded irrelevant studies. The full-text of potentially eligible studies for accuracy data was retrieved and carefully analyzed. The data collected by two independent researchers (K. Chen and A. A. Malik) were compared, and any comparative discrepancies were resolved by mutual consensus.

### Inclusion Criteria

We included full-text, peer-reviewed, cross-sectional, randomized controlled, and case-control studies using NAAT to diagnose and compare BSI with a culture reference standard. For the index test, the studies explicitly provided True positive (TP), True negative (TN), False positive (FP) and False negative (FN) values or included sufficient information to derive 2 × 2 contingency tables. All studies that met the standard BSI definition were eligible for inclusion, including fever, chills, palpitations, rapid breathing, gastrointestinal symptoms, confusion and shock.

### Exclusion Criteria

Studies were considered for exclusion if they were conference proceedings, commentaries, reviews, meta-analysis, editorials, case reports, mechanism, and animal experimentation. Studies providing insufficient data for constructing a 2 × 2 contingency table and comprising <10 specimens were excluded. Non-interpretable test results by both index test and microbiological reference standard were also not included.

### Data Extraction

Two independent analysts (K. Chen and A. A. Malik) scanned all the related papers with pre-specified eligibility requirements in order to ensure the reproducibility of study selection. Disagreed studies were resolved by consultation with a third investigator (S. C. Ojha). The data were collected from eligible studies including authorship, publishing year, country, settings, study type, patient selection, patient characteristics, sample type, sample size, NAAT specifics, potential risks, and information for construction of 2 × 2 contingency table. For missing details, ambiguous reference standards, and specimen preparation techniques, the authors were consulted individually. Contingency tables for NAAT performance compared to microbiological reference standards were constructed on the basis of available data from the qualifying studies. Studies involving different index tests as compared to the specific reference standard were considered separate studies.

### Quality Assessment

The methodological quality of the studies was assessed using the Quality Assessment of Diagnostic Accuracy Studies (QUADAS-2), a validated diagnostic study tool (27). The four QUADAS-2 domains were: patient selection, index test, reference standard, and flow and timing. All four domains for the possible risk of bias and the first three domains for applicability concerns were evaluated by reviewing authors (K. Chen and A. A. Malik). Each domain was assessed in terms of risk of bias using signaling questions that are answered with “yes,” “no,” or “unclear” and are judged as “low,” “high,” or “unclear,” respectively. The first three domains are simultaneously evaluated in terms of applicability concerns, which are also graded as “low,” “high,” or “unclear” with identical characteristics. The differences between the reviewing authors were settled by consensus.

### Statistical Analysis

The following software was used for data analysis: RevMan 5.4 (Nordic Cochrane Centre, Copenhagen, Denmark) for the methodological quality assessment of included studies and summary plots generation (28). Meta-DiSc 1.4 (Cochrane Colloquium, Barcelona, Spain) for computation of pooled summary estimates including specificity, sensitivity, likelihood ratios, diagnostic odds ratio (DOR), and heterogeneity amongst data (29). Diagnostic accuracy of NAAT in association with 95% CI was computed against microbiological culture using a random-effects model. Furthermore, the *I*-square (*I*^2^) statistics were used to evaluate the heterogeneity of the included studies, where *I*^2^ values ranging from 0 to 40% indicate low heterogeneity, 30–60% moderate heterogeneity, 50–90% substantial heterogeneity, and values >90% signify considerable heterogeneity (30). Using subgroup analysis, different specimen conditions, patient selection, study design, and patient type were analyzed as possible heterogeneity sources. Publication bias was inspected using Deeks' funnel plot asymmetry test (31). A *P*-value of <0.05 was generally considered to be statistically significant.

## Results

### Literature Selection

**Figure 1:**
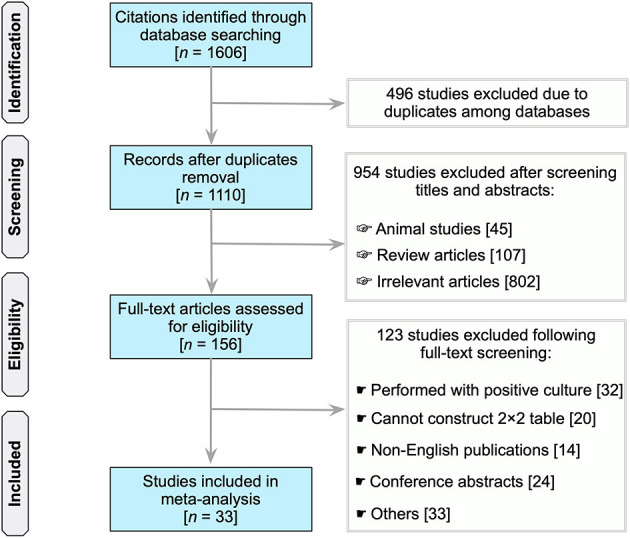
Flow chart of study selection.

In total, our search identified 1,606 unique records (PubMed, 402; Scopus, 818; Embase, 180; Web of Science, 206) (Figure 1). Of which, due to duplication in databases, 496 citations were eliminated. Following the scanning of the titles and abstracts of 1,110 publications, 156 studies considered potentially significant were subjected to a full-text revision. Supplementary Table 1 summarizes the studies reviewed, includin why these studies were excluded (see Supplementary Table 1). Essentially, 33 studies were included in subsequent analyses that met all the inclusion criteria (21, 32–63).

### Characteristics of the Included Studies

Table 1 shows the baseline features of the studies included (21, 32–63). Twenty-six studies were performed in countries with high incomes and seven in countries with lower-middle incomes. Studies reporting the usefulness of multiple index tests against a single reference standard were treated as separate studies. Centered upon this theory, this meta-analysis included 33 publications comprising 35 datasets. Out of 23 studies, 24 datasets (*n* = 8,547) evaluated the accuracy of NAAT for MSSA detection, while three studies (*n* = 479) examined the NAAT accuracy for MRSA detection in adults. Similarly, eight studies (*n* = 4,089) evaluated NAAT's accuracy for the identification of BSI in children. The total number of samples submitted for diagnostic assessment ranged from 15 to 2,844, with a median value of 100. All experimental tests were carried out in tertiary care hospitals or university research facilities. Only studies published in English before November 23, 2020, were included.

**Table 1:** Baseline features of included studies.

**References**	**Year**	**Country**	**Setting**	**Pros ** ** enroll**	**Patients selection**	**Patient type**	**Sample type**	**Sample size**	**NAAT specifics**	**Potential risks**
Abd El-Aziz et al. (32)	2020	Egypt	TCC	No	Convenience	Pediatric	Fresh	30	qPCR	Sepsis
Arabestani et al. (33)	2014	Iran	UHL	No	Convenience	Adult	Fresh/Frozen	126	mPCR	Bacteremia
Bloos et al. (34)	2010	Germany	TCC	Yes	Convenience	Adult	Fresh/Frozen	347	Septi*F*ast	Sepsis
Etchebarne et al. (35)	2017	USA	TCC	Yes	Consecutive	Adult	Fresh	31	LAMP	Sepsis
Faraji et al. (36)	2018	Iran	UHL	No	Convenience	Adult	Fresh	20	qPCR	IE
Fernández-Romero et al. (37)	2014	Spain	UHL	Yes	Convenience	Adult	Fresh	96	Septi*F*ast	BSI, SIRS
García-Gudiño et al. (38)	2018	Mexico	TCC	No	Convenience	Pediatric	Fresh/Frozen	22	PCR-DGGE	Sepsis
Ginn et al. (39)	2017	Australia	TCC	No	Convenience	Adult	Fresh/Frozen	15	MT-PCR	Sepsis
Grosse-Onnebrink et al. (40)	2017	Germany	UHL	Yes	Convenience	Adult	Fresh	72	Septi*F*ast	CF
Josefson et al. (41)	2011	Sweden	UHL	Yes	Consecutive	Adult	Fresh/Frozen	1540	Septi*F*ast	BSI
Kitagawa et al. (42)	1996	Japan	UHL	No	Convenience	Adult	Fresh	41	Nested PCR	Bacteremia
Knabl et al. (43)	2016	Austria	UHL	Yes	Consecutive	Adult	Fresh	58	Septi*F*ast	SIRS
Korber et al. (44)	2017	Austria	TCC	No	Convenience	Adult	Fresh	470	Septi*F*ast	Sepsis
Lehmann et al. (45)	2009	Germany	UHL	No	Convenience	Adult	Fresh	467	Septi*F*ast	Sepsis
Liberto et al. (46)	2006	Italy	TCC	No	Convenience	Adult	Fresh	31	qPCR-M	Bacteremia
Liu et al. (47)	2017	China	TCC	No	Convenience	Adult	Fresh	30	qPCR	Sepsis
Lucignano et al. (48)	2011	Italy	TCC	No	Convenience	Pediatric	Fresh	1,673	Septi*F*ast	Sepsis
Makhoul et al. (21)	2005	Israel	TCC	Yes	Convenience	Pediatric	Fresh	215	PCR	Bacteremia
Moore et al. (49)	2018	Uganda	TCC	No	Convenience	Adult	Fresh/Frozen	336	qPCR-TAC	Sepsis
Obara et al. (50)	2011	Japan	UHL	Yes	Convenience	Adult	Fresh	78	Septi*F*ast	Bacteremia
Oeser et al. (51)	2020	UK	TCC	No	Convenience	Pediatric	Fresh/Frozen	208	qPCR	Sepsis
Pasqualini et al. (52)	2012	Italy	UHL	Yes	Consecutive	Adult	Fresh	391	Septi*F*ast	SIRS
Peters et al. (53)	2007	Netherlands	UHL	No	Convenience	Adult	Fresh/Frozen	175	qPCR	Bacteremia
Rogina et al. (54)	2014	Slovenia	TCC	No	Consecutive	Adult	Fresh	23	SeptiTest, IHP	SIRS, Sepsis
Santolaya et al. (55)	2011	Chile	TCC	Yes	Convenience	Pediatric	Fresh/Frozen	177	RT-PCR	Bacteremia
Schaub et al. (56)	2014	Switzerland	UHL	Yes	Convenience	Adult	Fresh/Frozen	110	Septi*F*ast	SIRS, Sepsis
Van den Brand et al. (57)	2018	Netherland	UHL	Yes	Convenience	Pediatric	Fresh/Frozen	91	mPCR	Sepsis
Wallet et al. (58)	2010	France	TCC	Yes	Consecutive	Adult	Fresh	100	Septi*F*ast	Sepsis
Wu et al. (59)	2011	Italy	TCC	No	Convenience	Pediatric	Fresh	1,673	mPCR	Sepsis
Xiao et al. (60)	2019	China	UHL	No	Consecutive	Adult	Fresh/Frozen	2,844	PCR-MCA	BSI
Yanagihara et al. (61)	2010	Japan	UHL	Yes	Convenience	Adult	Fresh/Frozen	407	Septi*F*ast	SIRS
Zboromyrska et al. (62)	2016	Spain	TCC	Yes	Convenience	Adult	Fresh	92	GeneXpert	CRB
Ziegler et al. (63)	2016	Sweden	UHL	No	Consecutive	Adult	Fresh/Frozen	696	MST	Sepsis

*BSI, bloodstream infection; CRB, catheter related bacteremia; IE, infective endocarditis; IHP, in-house PCR; pros enroll, prospective enrollment; MST, magicplex sepsis real-time test; MT-PCR, multiplexed tandem real-time PCR; PCR-MCA, PCR coupled with melting curve analysis; qPCR, quantitative PCR; SIRS, systemic inflammatory response syndrome; TCC, tertiary care center (hospital); UHL, university hospital laboratory*.

### Quality Appraisal

**Figure 2:**
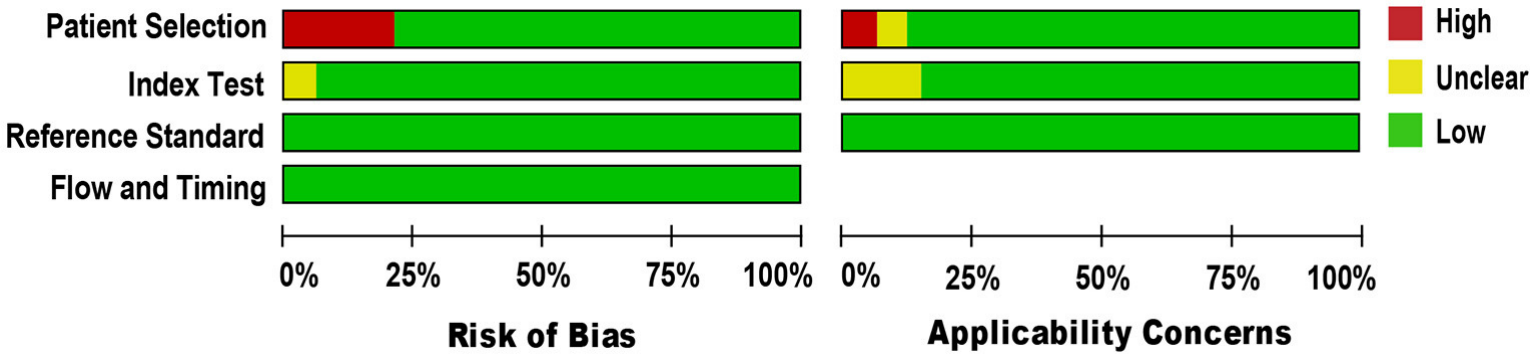
Methodological quality and risk of bias assessment of the eligible studies.

We applied the QUADAS-2 tool to evaluate the methodological quality of included studies against microbiological culture reference standard (see Figure 2). For a few studies in the patient selection domain (34, 38, 40, 48, 53, 55, 63), the risk of bias was high because the studies could not prevent improper sample exclusion. Regarding the applicability concern, all studies included blood samples from patients suspected of BSI, indicating a low risk of bias. Other domains, including the index test and the reference standard, were presumably at low risk of bias, as NAAT used pre-established binary response investigation criteria. Concerns about the index test conduct's applicability are unclear, as no proven protocol is available for global use. Reference standards of all studies have been carried out in hospitals or university-affiliated reference labs; as such, we expect operator error bias to be of low concern. Subsequently, there was no uncertainty regarding the possible risk of bias in the flow and timing domain as both the index test and the reference standards were applied to the same samples. In general, the quality of the studies included in our meta-analysis met the methodological standards.

### Summary Estimates

The studies were rather heterogeneous, so obtaining NAAT pooled summary estimates from integrated pediatric and adult populations was not considered meaningful for antimicrobial therapy. Initially, we concentrated on diagnosing BSI in children as it is often difficult to procure sufficiently large amounts of blood from children for microbiological culture. Subsequently, the pooled summary estimates of NAAT in the adult population to diagnose BSI were assessed. The accuracy of index tests, commercial tests, and a potential source of heterogeneity among studies in predefined subgroups have also been demonstrated.

### Detection of BSI Among the Pediatric Population

**Figure 3:**
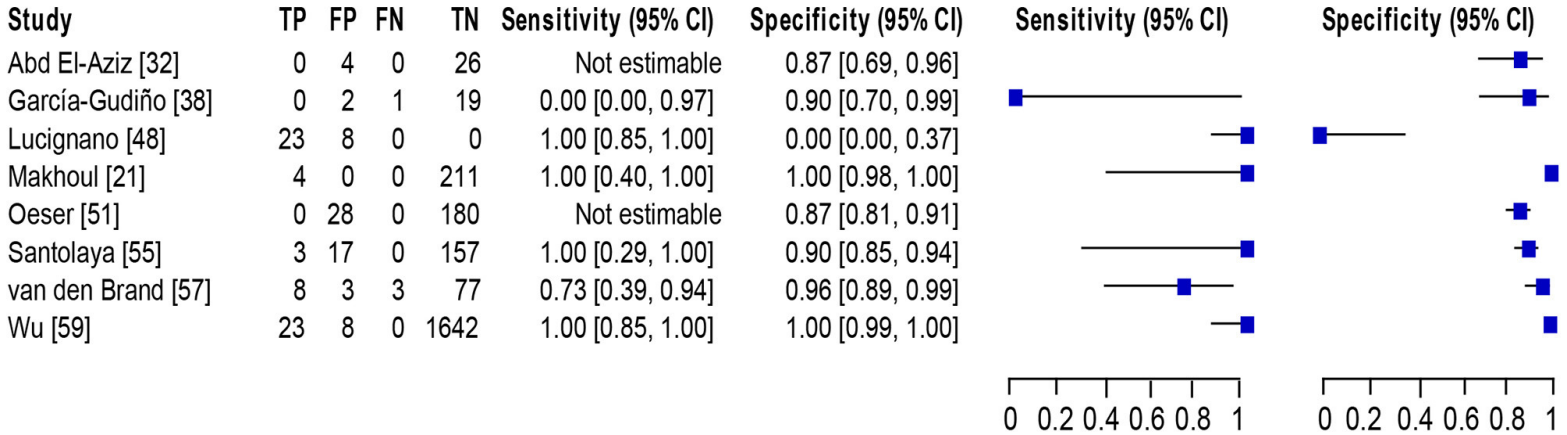
Forest plot for detection of MSSA in the pediatric population. The square stands for the sensitivity and specificity of a particular study, the black line represents its confidence interval. TP, true positive, FP, false positive, FN, false negative, TN, true negative, CI, confidence interval.

Eight studies (21, 32, 38, 48, 51, 55, 57, 59) met the inclusion criteria for comparing NAAT with a microbiological culture among children consisting of a total of 4,089 samples for MSSA detection in suspected BSI patients. The sensitivity and specificity of NAAT for MSSA detection ranged from 0.25 (95% CI 0.0–0.94) to 1.0 (95% CI, 0.85–1.0), whilst our search did not result in any MRSA studies (Figure 3). The pooled summary estimates of NAAT for detection of MSSA in blood were [sensitivity: 0.89 (95% CI 0.76–0.96), specificity: 0.98 (95% CI 0.97–0.98), positive likelihood ratio (PLR): 26.9 (95% CI 6.35–114.2), negative likelihood ratio (NLR): 0.25 (95% CI 0.08–0.8), DOR: 142.34 (95% CI 16.85–1,202.7)]. The *I*^2^ sensitivity and specificity statistical values were 57.0 and 95.7%, respectively, indicating substantial to considerable heterogeneity. The area under the curve (AUC) of summary receiver operating characteristics (SROC) was 0.90 (95% CI 0.74–1.0), indicating overall justifiable diagnostic validity (**Figure 5A**).

### Detection of BSI Among the Adult Population

**Figure 4:**
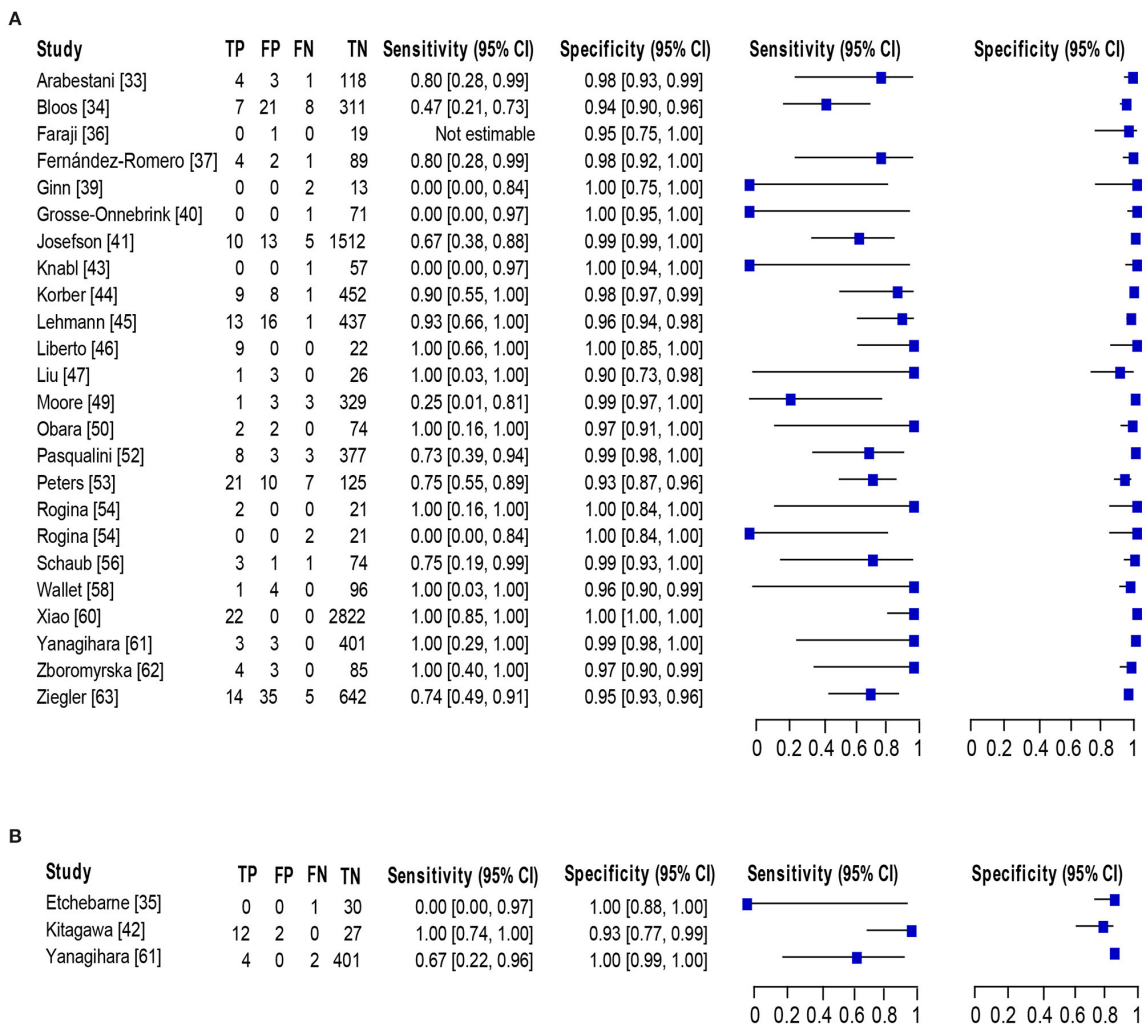
Forest plot for detection of **(A)** MSSA and **(B)** MRSA in the adult population. The square stands for the sensitivity and specificity of a particular study, the black line represents its confidence interval. TP, true positive, FP, false positive, FN, false negative, TN, true negative, CI, confidence interval.

**Figure 5:**
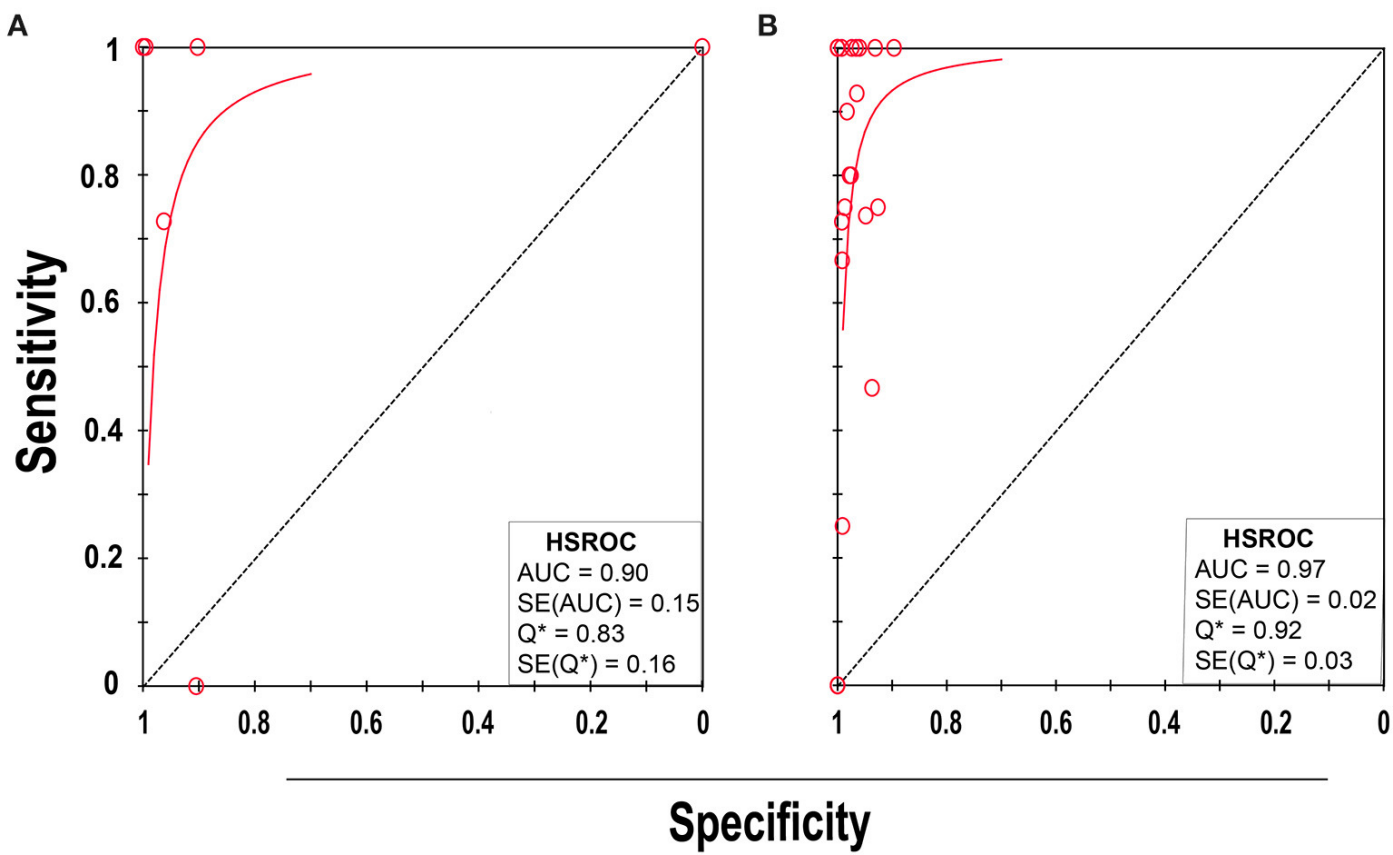
SROC plot of NAAT for **(A)** pediatric and **(B)** adult population. Red circles indicate the data point from each of the investigations, and the solid blue line represents the SROC curve.

Of 23 studies (33–37, 39–47, 49, 50, 52–54, 56, 58, 60–63), 24 datasets consisting of 8,547 blood samples evaluated the accuracy of NAAT for MSSA identification, whereas three datasets (*n* = 479) assessed the NAAT accuracy for MRSA identification. The sensitivity and specificity of NAAT for identification of MSSA ranged from 0.17 (95% CI 0.0–0.82) to 1.0 (95% CI 0.85–1.00) and from 0.90 (95% CI 0.73–0.98) to 1.00 (95% CI 0.99–1.00), respectively (Figure 4A). While the sensitivity and specificity of NAAT for identification of MRSA ranged from 0.25 (95% CI 0.0–0.94) to 1.0 (95% CI 0.74–1.00) and from 0.93 (95% CI 0.77–0.99) to 1.00 (95% CI 0.99–1.0), respectively (Figure 4B). Pooled summary estimates of NAAT for MSSA identification was lower [sensitivity: 0.76 (95% CI 0.69–0.82), specificity: 0.98 (95% CI 0.98–0.99), PLR: 28.63 (95% CI 18.59–44.1), NLR: 0.34 (95% CI 0.23–0.50), DOR: 116.38 (95% CI 57.68–234.8)] compared to MRSA [sensitivity: 0.83 (95% CI 0.59–0.96), specificity: 0.99 (95% CI 0.98–1.0), PLR: 40.73 (95% CI 3.89–426.1), NLR: 0.32 (95% CI 0.08–1.32), DOR: 268.6 (95% CI 32.1–2250.0)]. The *I*^2^ statistical scores for sensitivity and specificity of MSSA identification were 57.4 and 88.9%, respectively, indicating substantial to considerable heterogeneity. While *I*^2^ statistical scores for sensitivity and specificity of MRSA were 76.9 and 82.1%, indicating considerable heterogeneity. The AUC of SROC for assorted MSSA and MRSA among adult population was 0.97 (95% CI 0.94–1.0), suggesting overall valid diagnostic accuracy (Figure 5B).

### Diagnostic Accuracy of In-house vs. Commercial Tests

The diagnostic accuracy of studies based on various NAA tests is summarized in Table 2. For pediatric and adult populations, the pooled summary estimates of the in-house NAA tests for detecting MSSA were consistently higher (sensitivity ≥78%; specificity ≥93%). The pooled summary estimates of the commercial tests were higher for MSSA identification [sensitivity: 0.75 (95% CI 0.66–0.82), specificity: 0.98 (95% CI 0.97–0.98), PLR: 31.9 (95% CI 20.4–50.0), NLR: 0.3 (95% CI 0.2–0.5), DOR: 126.1 (95% CI 62.1–256.2) and AUC 0.98 (95% CI 0.96–0.99)] as compared to MRSA [sensitivity: 0.56 (95% CI 0.21–0.88), specificity: 1.0 (95% CI 0.99–1.0), PLR: 113.2 (95% CI 4.7–2,699.8), NLR: 0.53 (95% CI 0.26–1.0), and DOR: 234.2 (95% CI 4.65–11,801.6)]. Among NAAT studies, Septi*F*ast were consistently performed to detect BSI in blood samples compared to other tests.

**Table 2:** Subgroup analysis of studies based on different NAA tests.

**Subject**	**Diagnostic target**	**Subgroup**	**Total data**	**NAAT methods**	**% Sensitivity (95% CI)**	**% Specificity (95% CI)**	**PLR (95% CI)**	**NLR (95% CI)**	**DOR (95% CI)**	**AUC (95% CI)**
Adult	*S. aureus*	In-house	8		79 (66–88)	100 (99–100)	23.4 (6.5–84.7)	0.3 (0.1–0.9)	91.7 (12.3–684.4)	86 (84–88)
				qPCR (3)	75 (56–89)	92 (88–96)	9.3 (5.3–16.2)	0.3 (0.2–0.5)	34.7 (12.8–93.8)	94 (92–97)
				PCR (2)	56 (20–88)	98 (94–100)	28.3 (9.2–87.2)	0.4 (0.1–3.0)	67.6 (6.75–677.9)	–
				PCR-MCA (1)	100 (85–100)	100 (100–100)	–	0.0	–	100 (99–100)
				MT-PCR (1)	0 (0–84)	100 (75–100)	–	1.0 (1.0–1.0)	–	87 (60–98)
		Commercial	17		75 (66–82)	98 (97–98)	31.9 (20.4–50.0)	0.3 (0.2–0.5)	126.1 (62.1–256.2)	98 (96–99)
				Septi*F*ast (14)	76 (66–84)	98 (98–99)	36.9 (21.9–62.4)	0.3 (0.2–0.5)	160.8 (66.8–387.2)	99 (97–100)
				qPCR TAC (1)	25 (63–81)	99 (97–100)	27.7 (3.6–212.2)	0.8 (0.4–1.3)	–	98 (96–99)
				GeneXpert (1)	100 (40–100)	97 (90–99)	29 (9.7–89.2)	0.0	–	97 (91–99)
				MST (1)	74 (49–91)	95 (93–96)	14.3 (9.4–21.7)	0.3 (0.1–0.6)	–	94 (92–96)
	MRSA	In-house	1	Nested PCR (1)	100 (74–100)	93 (77–99)	14.5 (3.8–55.2)	0.0	–	95 (84–99)
		Commercial	2		56 (20–88)	100 (99–100)	113 (4.7–2699.8)	0.53 (0.26–1.0)	234 (4.65–11801.6)	–
				LAMP (1)	0 (0–98)	100 (88–100)	–	1.0 (1.0–1.0)	–	97 (83–100)
				Septi*F*ast (1)	67 (22–96)	100 (99–100)	–	0.3 (0.1–1.0)	–	100 (98–100)
Children	*S. aureus*	In-house	7		78 (57–92)	96 (95–97)	17.5 (5.0–61.8)	0.4 (0.2–0.8)	72.2 (9.9–528.0)	74 (57–97)
				qPCR (4)	75 (48–93)	89 (86–92)	9.1 (4.8–17.4)	0.3 (0.2–0.7)	39.7 (10.4–151.2)	93 (83–100)
				GSPBRT-PCR (1)	100 (29–100)	100 (99–100)	–	0.0	–	100 (99–100)
				PCR-DGGE (1)	0 (0–98)	91 (70–99)	0.0	1 (0.9–1.3)	–	86 (65–97)
				PCR (1)	100 (40–100)	100 (98–100)	–	0.0	–	–
		Commercial	1	Septi*F*ast (1)	100 (85–100)	100 (99–100)	206 (103–412)	0.0	–	100 (99–100)

*not estimable; AUC, area under curve; DOR, diagnostic odds ratio; LAMP, loop-mediated isothermal amplification; NAAT, nucleic acid amplification tests; NLR, negative likelihood ratio; PLR, positive likelihood ratio; qPCR, quantitative PCR*.

### Meta-Regression and Subgroup Analysis

We assessed the possible source of heterogeneity through a meta-regression analysis on predefined subgroups. Meta-regression suggested that study design (prospective/others), country (developed/developing), and patient selection (consecutive/convenience) were not the significant source of heterogeneity (meta-regression *P* = 0.42, *P* = 0.34, and *P* = 0.62, respectively) with the exception of the sample condition (fresh/frozen) (*P* = 0.04).

### Publication Bias

Using Deek's funnel plot asymmetry test, publication bias was measured. In our analysis, we did not find striking publication bias (*P* = 0.25).

## Discussion

Despite advances in supportive care, BSI is a leading cause of death worldwide (10). In recent years, most studies have centered on screening positive blood culture to identify causative staphylococcal pathogen (64–66); however, it remains a matter of concern as every hour of delay in initiating effective antimicrobial therapy increases mortality by 7.6% in sepsis patients (67). Therefore, it is essential to recognize staphylococcal species and their resistance markers rapidly in patients with suspected BSI, as the prompt intervention will lead to improved clinical outcomes with effective antimicrobial therapy. Currently, several multiplex molecular assays have been cleared by the Food and Drug Administration (FDA) that can identify a wide range of microorganisms concurrently with specific resistance genes in blood samples, and several of these assays have been rendered commercially accessible (68). Nevertheless, the numerous case descriptions and the diverse samples used in the various studies make it difficult to compare research results and restrict diseases' management. We, therefore, conducted a systematic review and meta-analysis to evaluate NAAT's diagnostic performance for diagnosing BSI in clinically suspected patients.

**Figure 6:**
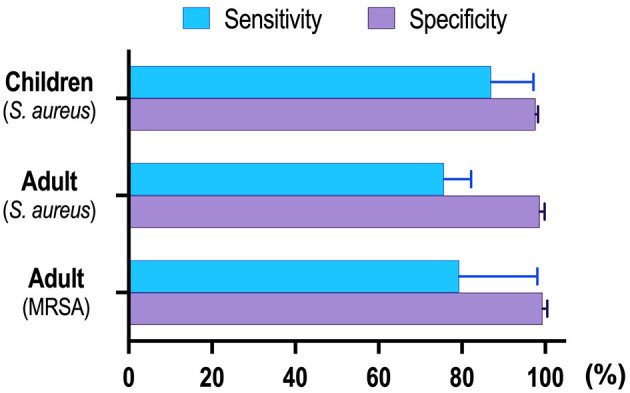
Summary for NAAT's pooled sensitivity and specificity.

Our results revealed that NAAT overall summary estimates for MRSA detection [sensitivity: 0.83 (95% CI 0.59–0.96), specificity: 0.99 (95% CI 0.98–1.0), AUC: 0.98 (95% CI 0.96–0.99)] were higher as compared to MSSA [sensitivity: 0.76 (95% CI 0.69–0.82), specificity: 0.98 (95% CI 0.98–0.99), AUC: 0.98 (95% CI 0.97–0.99)] among adults. Relatively smaller sample size, different DNA extraction technique, target genes adopted, and reaction material quality are among factors that could have contributed to greater NAAT accuracy for MRSA detection. Figure 6 shows the pooled sensitivity and specificity of NAAT for BSI detection in children and adults.

In the pediatric population, NAAT displayed consistently higher diagnostic accuracy for MSSA detection [sensitivity: 0.89 (95% CI 0.76–0.96), specificity: 0.98 (95% CI 0.97–0.98), AUC: 0.90 (95% CI 0.74–1.0)]. Albeit not perfect, the higher sensitivity of NAAT among a pediatric population, where the acquisition of large volumes of blood is a major concern, and relatively small non-interpretable results, encourages the use of the test for the diagnosis of BSI in principle. Furthermore, NAAT identified more pathogens than blood cultures alone in all studies, and specificity (~98%) of the test was consistent among pediatric and adult populations, highlighting higher NAAT diagnostic accuracy. Compared to previously published systematic reviews conducted by Pammi et al. (69) in the neonatal population, we found that the pooled sensitivity of 0.90 (95% CI 0.82–0.95) and specificity of 0.93 (95% CI 0.89–0.96) were similar to our study. Similarly, a meta-analysis by Su et al. (70) mainly summarized the diagnostic value of the 16S rRNA gene PCR test for neonates, which reported a nearly similar sensitivity of 0.85 (95% CI, 0.81–0.88) and a specificity of 0.96 (95% CI, 0.95–0.96) for BSI diagnosis. All these reviews were not unique to MSSA or MRSA for the definitive diagnosis of BSI.

Additionally, NAAT subgroup analysis found that in-house tests [sensitivity: 0.78 (95% CI 0.66–0.88), specificity: 0.99 (95% CI 0.99–1.0)] and commercial tests [sensitivity: 0.75 (95% CI 0.66–0.82), specificity: 0.98 (95% CI 0.97–0.98)] were comparable for MSSA detection (Table 2). The PLR for commercial research was 31.9, implying that patients with BSI are ~32 times more likely than patients without BSI to be NAA test positive. In the case of MRSA, there was insufficient data in the in-house and commercial test subgroups to allow meaningful comparisons. Searching for more accurate commercial test details, we observed that Septi*F*ast was often used in BSI diagnosis, and the pooled summary estimates of tests [sensitivity: 0.76 (95% CI 0.66–0.84), specificity: 0.98 (95% CI 0.98–0.99)] corresponded well-with the study by Chang and colleagues [sensitivity: 0.75 (95% CI 0.65–0.83), specificity: 0.92 (95% CI 0.90–0.95)] (71). However, Chang and colleagues evaluated the accuracy of Septi*F*ast against a composite reference standard, including bacteremia and fungemia, which was not unique to BSI.

The vital strengths of this study include a rigorous search strategy, utility of systemic guidelines, impartial selection criteria, a precise reference standard, a bivariate random-effects model for data-manipulation, meta-regression analysis on predefined subgroups, and independent analysts' interpretation. Studies that did not adhere to specific guidelines for diagnosing BSI, as well as those with a high risk of bias and high applicability concerns, as judged by the QUADAS-2 tool, were removed from subsequent analysis. We also excluded studies with fewer than ten samples to reduce the effects of publication bias while also discouraging future researchers from conducting small-scale studies, which is consistent with other meta-analyses (72, 73). Furthermore, and involved pre-enrichment steps before molecular testing, which may tend to overstate the index test's diagnostic performance, were excluded.

There are a few limitations to our analysis. It is likely that we may have overlooked a few crucial studies through systematic literature searches across databases. Due to the high degree of reporting variability of the included studies, the effect of factors such as sample volume, non-standardized sample preparation, NAA testing expertise, amplification procedures, and laboratory facilities on the accuracy of NAA tests could not be addressed. It should be noted that the gene targets were also different, which could be possible reasons for the heterogeneity. Although the study design, sample condition, and patient selection were not significant sources of heterogeneity in the meta-regression analysis, these variables could enhance heterogeneity and limit the generalizability of the NAAT's overall diagnostic accuracy. In addition, this meta-analysis was constrained due to insufficient MRSA studies among both children and adult populations, and should be interpreted with caution.

## Conclusions

Our findings suggest that currently available molecular assays may not have adequate diagnostic accuracy to replace microbial cultures. However, molecular assays have a shorter turnaround time, a higher proportion of false-positives, a higher specificity, and may detect minute amounts of DNA from a dead organism. Furthermore, because the specimen type and gene target for MRSA detection are the same in adults and children, NAAT's accuracy in adult blood samples could provide a glimpse of its applicability for subsequent detection in the pediatric population. Therefore, NAAT should be considered as the preferred initial tests for diagnosing BSI in order to avoid unnecessary anti-MRSA therapy. The utility of NAAT, in combination with microbiological culture, should be considered whenever feasible. Given the limited data on MRSA, it would be of interest to conduct a detailed investigation using a higher number of prospective studies to fully validate the clinical outcomes associated with NAAT's utility. Additionally, future research should analyze additional measures, including NAAT's impact on decreased hospitalizations, cost-effectiveness, antibiotic-related adverse effects, and electronic clinical decision supporting tools to accelerate therapy adjustment.

## Data Availability Statement

The original contributions presented in the study are included in the article/Supplementary Material, further inquiries can be directed to the corresponding author/s.

## Author Contributions

SCO conceptualized the study. KC, AAM, and SCO performed the literature search, analyzed data, and drafted the manuscript. Y-JS, SA, CS, C-LD, and SCO reviewed and edited the manuscript. All authors contributed to the article and approved the submitted version.

## Conflict of Interest

The authors declare that the research was conducted in the absence of any commercial or financial relationships that could be construed as a potential conflict of interest.

## Publisher's Note

All claims expressed in this article are solely those of the authors and do not necessarily represent those of their affiliated organizations, or those of the publisher, the editors and the reviewers. Any product that may be evaluated in this article, or claim that may be made by its manufacturer, is not guaranteed or endorsed by the publisher.
