# Supplementary material for: Clinical Utility of Molecular Tests for Guiding Therapeutic Decisions in Bloodstream Staphylococcal Infections: A Meta-Analysis
Source: Front Pediatr. 2021 Aug 5;9:713447. doi: 10.3389/fped.2021.713447 (PMC8374148; doi:10.3389/fped.2021.713447)
Supplement: Supplementary file 1 [file Table_1.DOCX]

**Table S1** Details of all the publications that were deemed relevant, and hence, fully screened by the first two authors (*n* = 156). Reasons why studies have been excluded are given (*n* = 123). Studies included in our meta-analysis are shaded with light yellow color (*n* = 33).

| First author [reference] | Inclusion (I)  /Exclusion (E) | Reasons |
| --- | --- | --- |
| Abd El-Aziz (1) | I |  |
| Adiku (2) | E | Cannot construct 2×2 contingency table |
| Afsharpaiman (3) | E | Cannot construct 2×2 contingency table |
| Al-Zahrani (4) | E | Cannot construct 2×2 contingency table |
| Amin-Desai (5) | E | Conference abstract |
| Arabestani (6) | I |  |
| Aubert (7) | E | Non-English publication |
| Bacconi (8) | E | Measurement of PCR amplicon via mass spectroscopy |
| Bauer (9) | E | Performed with positive blood culture |
| Bernaschi (10) | E | Conference abstract |
| Besharati (11) | E | Characterization of isolated strains |
| Bianco (12) | E | Performed with positive blood culture |
| Bloos (13) | I |  |
| Brown (14) | E | Conference abstract |
| Burrack-Lange (15) | E | Performed with positive blood culture |
| Canning (16) | E | Associated with enzymatic assay |
| Carlesse (17) | E | Performed with incubated blood culture |
| Carroll (18) | E | Performed with positive blood culture |
| Carter (19) | E | Not specific to staphylococcal bacteremia |
| Chan (20) | E | Cannot construct 2×2 contingency table |
| Chelliah (21) | E | Performed with positive blood culture |
| Chen (22) | E | Conference abstract |
| Choi (23) | E | Hybridization assay with positive blood culture |
| Clerc (24) | E | Performed with positive blood culture |
| De Angelis (25) | E | Performed with improved diagnostic assay |
| Dekker (26) | E | Characterization of isolated strains |
| Delerue (27) | E | Performed with positive blood culture |
| Draz (28) | E | Cannot construct 2×2 contingency table |
| Drwiega (29) | E | Performed with positive blood culture |
| El Gawhary (30) | E | Performed with incubated blood culture |
| Emonet (31) | E | Performed with positive blood culture |
| Etchebarne (32) | I |  |
| Faraji (33) | I |  |
| Felsenstein (34) | E | Performed with positive blood culture |
| Fernandez-Cruz (35) | E | Evaluated persistence of blood culture positivity |
| Fernández-Romero (36) | I |  |
| Ferreira (37) | E | Non-English publication |
| Ferroni (38) | E | Non-English publication |
| Fitting (39) | E | Performed with positive blood culture |
| Fouad (40) | E | Conference abstract |
| Frye (41) | E | Performed with positive blood culture |
| Galindo-Sevilla (42) | E | Conference abstract |
| García-Gudiño (43) | I |  |
| Geiger (44) | E | Clinical consultation |
| Gholami (45) | E | Non-English publication |
| Gies (46) | E | No coagulase positive staphylococci detected |
| Gimeno (47) | E | Conference abstract |
| Ginn (48) | I |  |
| Gosiewski (49) | E | Not specific to staphylococcal bacteremia |
| Grosse-Onnebrink (50) | I |  |
| Guimaraes (51) | E | Evaluation of prognostic power of cell free DNA |
| Haag (52) | E | Cannot construct 2×2 contingency table |
| Haque (53) | E | Characterization of positive isolates |
| Hassanin (54) | E | Cannot construct 2×2 contingency table |
| Havaei (55) | E | Non-English publication |
| Hirvonen (56) | E | Conference abstract |
| Inglis (57) | E | Performed with positive blood culture |
| Istanbullu (58) | E | Could not recover MSSA OR MRSA in bloodstream |
| Josefson (59) | I |  |
| Kang (60) | E | Non-English publication |
| Kasper (61) | E | No coagulase positive staphylococci detected |
| Kelley (62) | E | Performed with positive blood culture |
| Khanes (63) | E | Conference abstract |
| Kim (64) | E | Conference abstract |
| Kitagawa (65) | I |  |
| Kitagawa (66) | E | Non-English publication |
| Knabl (67) | I |  |
| Korber (68) | I |  |
| Krulova (69) | E | Conference abstract |
| Lehmann (70) | I |  |
| Lehmann (71) | E | Development of an assay |
| Leikiem (72) | E | Performed with positive blood culture |
| Levi (73) | E | Performed with positive blood culture |
| Ley (74) | E | Cannot construct 2×2 contingency table |
| Liberto (75) | I |  |
| Liu (76) | I |  |
| Long (77) | E | Detection of cell-free DNA by NGS |
| Lucignano (78) | I |  |
| Mahanta (79) | E | Conference abstract |
| Makhoul (80) | I |  |
| Makhoul (81) | E | Cannot construct 2×2 contingency table |
| Mancilla (82) | E | Conference abstract |
| Mason (83) | E | Performed with positive blood culture |
| McHugh (84) | E | Performed with positive blood culture |
| Mehta (85) | E | Not staphylococcal bacteremia specific |
| Metwally (86) | E | Non-English publication |
| Midan (87) | E | Cannot construct 2×2 contingency table |
| Misawa (88) | E | Performed with positive blood culture |
| Mohamadi (89) | E | Cannot construct 2×2 contingency table |
| Mohammed (90) | E | Not staphylococcal bacteremia specific |
| Moore (91) | I |  |
| Moore (92) | E | Cannot construct 2×2 contingency table |
| Negoro (93) | E | Conference abstract |
| Nikolaras (94) | E | Characterization of culture positive isolates |
| Notario (95) | E | Non-English publication |
| Obara (96) | I |  |
| Oeser (97) | I |  |
| Ohlin (98) | E | Performed with positive blood culture |
| Orszag (99) | E | Not staphylococcal bacteremia specific |
| Orszag (100) | E | Conference abstract |
| Palomares (101) | E | Conference abstract |
| Papaparaskevas (102) | E | Non-English publication |
| Pasqualini (103) | I |  |
| Patel (104) | E | Conference abstract |
| Pavone (105) | E | Conference abstract |
| Perchorsky (106) | E | Performed with incubated blood culture |
| Peters (107) | I |  |
| Pingle (108) | E | Development of an assay |
| Pomorska-Wesołowska (109) | E | Confirmation of MRSA from positive isolates |
| Punjabi (110) | E | Performed with positive blood culture |
| Reier-Nilsen (111) | E | Not staphylococcal bacteremia specific |
| Reuter (112) | E | Performed with positive blood culture |
| Rogina (113) | I |  |
| Rohit (114) | E | PCR efficacy determined with enriched culture |
| Rozemeijer (115) | E | Conference abstract |
| Santolaya (116) | I |  |
| Sauget (117) | E | Cannot construct 2×2 contingency table |
| Scanvic (118) | E | Non-English publication |
| Schaub (119) | I |  |
| Šeputiene (120) | E | Development of an assay |
| Shachor-Meyouhas (121) | E | Cannot construct 2×2 contingency table |
| Shin (122) | E | Performed with positive blood culture |
| Silva-Junior (123) | E | Cannot construct 2×2 contingency table |
| Spencer (124) | E | Validation of Xpert with positive blood culture |
| Spreafico (125) | E | Conference abstract |
| Strålin (126) | E | Performed with PCR coupled with ESI-MS |
| Stranieri (127) | E | Performed with positive blood culture |
| Straub (128) | E | Cannot construct 2×2 contingency table |
| Swathirajan (129) | E | Cannot construct 2×2 contingency table |
| Tat Trung (130) | E | Evaluated for *Staphylococcus* spp. |
| Tong (131) | E | Non-English publication |
| Tröger (132) | E | Cannot construct 2×2 contingency table |
| Trung (133) | E | Conference abstract |
| Tsalik (134) | E | Conference abstract |
| Tumuhamye (135) | E | No comparative evaluation with NAAT |
| Turner (136) | E | Performed with positive blood culture |
| Ullrich (137) | E | Development of an assay |
| van de Groep (138) | E | Assay validation with positive blood culture |
| van den Brand (139) | E | No coagulase positive staphylococci detected |
| van den Brand (140) | I |  |
| Vincent (141) | E | Measurement of PCR amplicon via mass spectroscopy |
| Wallet (142) | I |  |
| Wang (143) | E | Performed with positive blood culture |
| Wang (144) | E | Conference abstract |
| Ward | E | Performed with positive blood culture |
| Wellinghausen (145) | E | Cannot construct 2×2 contingency table |
| Willey (146) | E | Conference abstract |
| Wolk (147) | E | Performed with positive blood culture |
| Wu (148) | E | Performed with positive blood culture |
| Wu (149) | I |  |
| Wu (150) | E | Non-English publication |
| Xiao (151) | I |  |
| Yanagihara (152) | I |  |
| Yue (153) | E | Non-English publication |
| Zboromyrska (154) | I |  |
| Zhang (155) | E | Cannot construct 2×2 contingency table |
| Ziegler (156) | I |  |

**REFERENCES**

1. Abd El-Aziz NK, Gharib AA, Mohamed EAA, Hussein AH. Real-time PCR versus MALDI-TOF MS and culture-based techniques for diagnosis of bloodstream and pyogenic infections in humans and animals. J Appl Microbiol. 2020.

2. Adiku TK, Asmah RH, Rodrigues O, Goka B, Obodai E, Adjei AA, et al. Aetiology of acute lower respiratory infections among children under five years in Accra, Ghana. Pathogens. 2015;4(1):22-33.

3. Afsharpaiman S, Mamishi S, Pourakbari B, Siyadati A, Tabatabaee P, Khotaee G. Diagnosis of bacteremia using universal PCR in febrile ill children. Acta Medica Iranica. 2007;45(2):131-8.

4. Al-Zahrani AK, Ghonaim MM, Hussein YM, Eed EM, Khalifa AS, Dorgham LS. Evaluation of recent methods versus conventional methods for diagnosis of early-onset neonatal sepsis. J Infect Dev Ctries. 2015;9(4):388-93.

5. Amin-Desai K, Ansari Z, Athanasiou P, Baeza M, Bauer R, Barr A, et al. Rapid, sensitive detection of clinical bloodstream infections directly from whole human blood. J Mol Diagn. 2016;18(6):984.

6. Arabestani MR, Fazzeli H, Esfahani BN. Identification of the most common pathogenic bacteria in patients with suspected sepsis by multiplex PCR. J Infect Dev Ctries. 2014;8(4):461-8.

7. Aubert G, Vautrin AC, Michel VP, Fresard A, Dorche G. [Evaluation of three automated blood culture systems. Bio Argod, Bact T/Alert, bactec NR-860]. Pathol Biol (Paris). 1993;41(4):434-40.

8. Bacconi A, Richmond GS, Baroldi MA, Laffler TG, Blyn LB, Carolan HE, et al. Improved sensitivity for molecular detection of bacterial and candida infections in blood. J Clin Microbiol. 2014;52(9):3164-74.

9. Bauer KA, West JE, Balada-Llasat JM, Pancholi P, Stevenson KB, Goff DA. An antimicrobial stewardship program's impact with rapid polymerase chain reaction methicillin-resistant *Staphylococcus aureus*/*S. aureus* blood culture test in patients with *S. aureus* bacteremia. Clin Infect Dis. 2010;51(9):1074-80.

10. Bernaschi P, Ranno S, Lucignano B, Pizzorno B, Liesenfeld O, Menichella D. Value of multiplex-PCR (SeptiFast) for the diagnosis of bacterial and fungal pathogens in newborns and children with suspected sepsis. Clin Microbiol Infect. 2010;16:S541.

11. Besharati R, Ghafouri M, Safamanesh S, Khosrojerdi M, Ghazvini K, Nojumi S, et al. Molecular epidemiology of panton-valentine leukocidin harboring hospital-associated methicillin-resistant *Staphylococcus aureus* in septicemic children, Northeastern Iran, Bojnurd. Jundishapur J Microbiol. 2019;12(2): e68183.

12. Bianco G, Boattini M, Iannaccone M, Sidoti F, Cavallo R, Costa C. Detection of antibiotic resistance genes from blood cultures: performance assessment and potential impact on antibiotic therapy management. J Hosp Infect. 2019;102(4):465-9.

13. Bloos F, Hinder F, Becker K, Sachse S, Dessap AM, Straube E, et al. A multicenter trial to compare blood culture with polymerase chain reaction in severe human sepsis. Intensive Care Med. 2010;36(2):241-7.

14. Brown J, Paladino J. Mortality and cost-effectiveness of rapid MRSA PCR testing in hospitalised patients with bacteraemia: A decision model. Clin Microbiol Infect. 2009;15:S57-S8.

15. Burrack-Lange SC, Personne Y, Huber M, Winkler E, Weile J, Knabbe C, et al. Multicenter assessment of the rapid unyvero blood culture molecular assay. J Med Microbiol. 2018;67(9):1294-301.

16. Canning B, Mohamed I, Wickramasinghe N, Swindells J, O'Shea MK. Thermonuclease test accuracy is preserved in methicillin-resistant *Staphylococcus aureus* isolates. J Med Microbiol. 2020;69(4):548-51.

17. Carlesse F, Cappellano P, Quiles MG, Menezes LC, Petrilli AS, Pignatari AC. Clinical relevance of molecular identification of microorganisms and detection of antimicrobial resistance genes in bloodstream infections of paediatric cancer patients. BMC Infect Dis. 2016;16(1):462.

18. Carroll KC, Reid JL, Thornberg A, Whitfield NN, Trainor D, Lewis S, et al. Clinical Performance of the novel GenMark Dx ePlex Blood Culture ID Gram-Positive Panel. J Clin Microbiol. 2020;58(4):e01730-19.

19. Carter K, Doern C, Jo CH, Copley LAB. The clinical usefulness of polymerase chain reaction as a supplemental diagnostic tool in the evaluation and the treatment of children with septic arthritis. J Pediatr Orthop. 2016;36(2):167-72.

20. Chan KYY, Lam HS, Cheung HM, Chan AKC, Li K, Fok TF, et al. Rapid identification and differentiation of Gram-negative and Gram-positive bacterial bloodstream infections by quantitative polymerase chain reaction in preterm infants. Crit Care Med. 2009;37(8):2441-7.

21. Chelliah A, Ravinder T, Katragadda R, Leela KV, Narayana Babu R. Isolation of MRSA, ESBL and AmpC - β -lactamases from neonatal sepsis at a tertiary care hospital. J Clin Diagnostic Res. 2014;8(6).

22. Chen X, Chih C, Hsu C, Chen P, Lee T, Teng L, et al. Rapid identification of pathogens from flagged blood cultures by multiplex PCR using the FilmArray system. J Infect Public Health. 2019;12(1):144.

23. Choi Y, Wang HY, Lee G, Park SD, Jeon BY, Uh Y, et al. PCR-reverse blot hybridization assay for screening and identification of pathogens in sepsis. J Clin Microbiol. 2013;51(5):1451-7.

24. Clerc O, Prod'hom G, Senn L, Jaton K, Zanetti G, Calandra T, et al. Matrix-assisted laser desorption ionization time-of-flight mass spectrometry and PCR-based rapid diagnosis of *Staphylococcus aureus* bacteraemia. Clin Microbiol Infect. 2014;20(4):355-60.

25. De Angelis G, Posteraro B, De Carolis E, Menchinelli G, Franceschi F, Tumbarello M, et al. T2Bacteria magnetic resonance assay for the rapid detection of ESKAPEc pathogens directly in whole blood. J Antimicrobial Chemother. 2018;73:20-6.

26. Dekker D, Wolters M, Mertens E, Boahen KG, Krumkamp R, Eibach D, et al. Antibiotic resistance and clonal diversity of invasive *Staphylococcus aureus* in the rural Ashanti Region, Ghana. BMC Infect Dis. 2016;16(1):720.

27. Delerue T, Cordel H, Delerue T, Figoni J, Dziri S, Billard-Pomares T, et al. Prediction of methicillin-resistant *Staphylococcus aureus* bloodstream infection: do we need rapid diagnostic tests? Eur J Clin Microbiol Infect Dis. 2019;38(7):1319-26.

28. Draz NI, Taha SE, Abou Shady NM, Abdel Ghany YS. Comparison of broad range 16S rDNA PCR to conventional blood culture for diagnosis of sepsis in the newborn. Egypt J Med Hum Genet. 2013;14(4):403-11.

29. Drwiega EN, Nichols KR, Israel EN, Knoderer CA. Impact of rapid mecA polymerase chain reaction rapid diagnostic testing for *Staphylococcus aureus* in a pediatric setting. Infect Dis Clin Pract. 2019;27(5):268-72.

30. El Gawhary S, El-Anany M, Hassan R, Ali D, El Gameel EQ. The role of 16S rRNA gene sequencing in confirmation of suspected neonatal sepsis. J Trop Pediatr. 2016;62(1):75-80.

31. Emonet S, Charles PG, Harbarth S, Stewardson AJ, Renzi G, Uckay I, et al. Rapid molecular determination of methicillin resistance in staphylococcal bacteraemia improves early targeted antibiotic prescribing: a randomized clinical trial. Clin Microbiol Infect. 2016;22(11):946.e9-.e15.

32. Etchebarne BE, Li Z, Stedtfeld RD, Nicholas MC, Williams MR, Johnson TA, et al. Evaluation of nucleic acid isothermal amplification methods for human clinical microbial infection detection. Front Microbiol. 2017;8(DEC).

33. Faraji R, Behjati-Ardakani M, Faraji N, Moshtaghioun SM, Kalantar SM, Pedarzadeh A, et al. Molecular diagnosis of bacterial definite infective endocarditis by real-time polymerase chain reaction. Cardiol Res. 2018;9(2):99-106.

34. Felsenstein S, Bender JM, Sposto R, Gentry M, Takemoto C, Bard JD. Impact of a rapid blood culture assay for Gram-positive identification and detection of resistance markers in a pediatric hospital. Arch Pathol Lab Med. 2016;140(3):267-75.

35. Fernandez-Cruz A, Marin M, Kestler M, Alcala L, Rodriguez-Creixems M, Bouza E. The value of combining blood culture and SeptiFast data for predicting complicated bloodstream infections caused by Gram-positive bacteria or candida species. J Clin Microbiol. 2013;51(4):1130-6.

36. Fernández-Romero N, Quiles I, Jiménez C, Oliva MOL, Rivas B, Mingorance J, et al. Use of multiplex PCR in diagnosis of bloodstream infections in kidney patients. Diagn Microbiol Infect Dis. 2014;80(2):93-6.

37. Ferreira LE, Dalposso K, Hackbarth BB, Gonçalves AR, Westphal GA, França PH, et al. Molecular panel for detection of sepsis-related microorganisms. Rev Bras Ter Intensiva. 2011;23(1):36-40.

38. Ferroni A. [Epidemiology and bacteriological diagnosis of paediatric acute osteoarticular infections]. Arch Pediatr. 2007;14 Suppl 2:S91-6.

39. Fitting C, Parlato M, Adib-Conquy M, Memain N, Philippart F, Misset B, et al. Dnaemia detection by multiplex PCR and biomarkers for infection in systemic inflammatory response syndrome patients. PLoS One. 2012;7(6):e38916.

40. Danzer M, Hofer K, Stabentheiner S, Süßner S, Pröll J, Gabriel C. Application of a novel multiplex real-time PCR assay for sensitive detection of bacterial contamination in platelet concentrates. Vox Sanguinis. 2010;99:11-2.

41. Frye AM, Baker CA, Rustvold DL, Heath KA, Hunt J, Leggett JE, et al. Clinical impact of a real-time PCR assay for rapid identification of staphylococcal bacteremia. J Clin Microbiol. 2012;50(1):127-33.

42. Galindo-Sevilla N, Ramírez-Ramírez A, Santillán R, Morales D, Segura-Cervantes E, Mancilla-Ramírez J. Identification of etiological agents of neonatal sepsis by pyrosequencing has less sensitivity than qPCR. J Matern Fetal Neonatal Med. 2014;27:162.

43. García-Gudiño I, Yllescas-Medrano E, Maida-Claros R, Soriano-Becerril D, Díaz NF, García-López G, et al. Microbiological comparison of blood culture and amplification of 16S rDNA methods in combination with DGGE for detection of neonatal sepsis in blood samples. Eur J Pediatr. 2018;177(1):85-93.

44. Geiger K, Brown J. Rapid testing for methicillin-resistant *Staphylococcus aureus*: Implications for antimicrobial stewardship. Am J Health-Syst Pharm. 2013;70(4):335-42.

45. Gholami A, Arabestani MR. Comparison of real-time PCR method and blood culture in diagnosis of septicemia. Tehran Univ Med J. 2016;73(11):784-90.

46. Gies F, Tschiedel E, Felderhoff-Müser U, Rath PM, Steinmann J, Dohna-Schwake C. Prospective evaluation of SeptiFast Multiplex PCR in children with systemic inflammatory response syndrome under antibiotic treatment. BMC Infect Dis. 2016;16:378.

47. Gimeno E, Sorli L, Abella E, Alvarez-Larran A, Horcajada JP, Garrigos L, et al. Molecular diagnosis of bacteriemia in patients with neutropenic febrile oncohematologic. J Clin Oncol. 2011;29(15):S9033.

48. Ginn AN, Hazelton B, Shoma S, Cullen M, Solano T, Iredell JR. Quantitative multiplexed-tandem PCR for direct detection of bacteraemia in critically ill patients. Pathology. 2017;49(3):304-8.

49. Gosiewski T, Jurkiewicz-Badacz D, Sroka A, Brzychczy-Włoch M, Bulanda M. A novel, nested, multiplex, real-time PCR for detection of bacteria and fungi in blood. BMC Microbiol. 2014;14:144.

50. Grosse-Onnebrink J, Stehling F, Tschiedel E, Olivier M, Mellies U, Schmidt R, et al. Bacteraemia and fungaemia in cystic fibrosis patients with febrile pulmonary exacerbation: A prospective observational study. BMC Pulm Med. 2017;17(1):96.

51. Guimaraes AO, Gutierrez J, Maskarinec SA, Cao Y, Hong K, Ruffin F, et al. Prognostic power of pathogen cell-free DNA in staphylococcus aureus bacteremia. Open Forum Infectious Diseases. 2019;6(4):ofz126.

52. Haag H, Locher F, Nolte O. Molecular diagnosis of microbial aetiologies using SepsiTest™ in the daily routine of a diagnostic laboratory. Diagn Microbiol Infect Dis. 2013;76(4):413-8.

53. Haque N, Taneja C, Oster G, Zervos M, Zilber S, Kyan PO, et al. Epidemiology of community-acquired and health care-associated *Staphylococcus aureus* pneumonia. Infect Dis Clin Pract. 2010;18(3):170-6.

54. Hassanin N, Abdallah NMA, Abdalla NEH, Kholeif L, Shabban M. Nested multiplex PCR for detection of bacterial and fungal blood stream infections in patients with hematological malignancies. J Infect Dev Ctries. 2020;14(5):511-8.

55. Havaei SA, Moghim S, Shahin M, Azimian A, Ghanbari F, Shokri D, et al. A comparison between polymerase chain reaction, oxacillin agar dilusion and cefoxitin disk diffusion methods in detection of methicillin resistance in *Staphylococcus aureus*. Journal of Isfahan Medical School. 2013;31:232.

56. Hirvonen JJ, Kaukoranta SS. Rapid detection of methicillin-sensitive and resistanct *Staphylococcus aureus* and methicillin-resistant coagulase-negative *Staphylococci* from blood cultures by automated PCR assay. BMC Proc. 2011;5:P204

57. Inglis TJJ, Bzdyl N, Chua IJ, Urosevic NM, Leung MJ, Geelhoed E. Improved blood culture identification by FilmArray in cultures from regional hospitals compared with teaching hospital cultures. J Med Microbiol. 2016;65(1):56-61.

58. İstanbullu K, Köksal N, Çetinkaya M, Özkan H, Yakut T, Karkucak M, et al. The potential utility of real-time PCR of the 16s-rRNA gene in the diagnosis of neonatal sepsis. Turk J Pediatr. 2019;61(4):493-9.

59. Josefson P, Strålin K, Ohlin A, Ennefors T, Dragsten B, Andersson L, et al. Evaluation of a commercial multiplex PCR test (SeptiFast) in the etiological diagnosis of community-onset bloodstream infections. Eur J Clin Microbiol Infect Dis. 2011;30(9):1127-34.

60. Kang J, Ma E, Fang J, Cui X. *Staphylococcus aureus* DNA in human venous blood detected by real-time quantitative PCR assay. Chinese J Clin Nutr. 2014;22(2):101-5.

61. Kasper DC, Altiok I, Mechtler TP, Böhm J, Straub J, Langgartner M, et al. Molecular detection of late-onset neonatal sepsis in premature infants using small blood volumes: Proof-of-concept. Neonatology. 2013;103(4):268-73.

62. Kelley PG, Grabsch EA, Farrell J, Xie S, Montgomery J, Mayall B, et al. Evaluation of the Xpert™ MRSA/SA blood culture assay for the detection of *Staphylococcus aureus* including strains with reduced vancomycin susceptibility from blood culture specimens. Diagn Microbiol Infect Dis. 2011;70(3):404-7.

63. Khanes G, Liutko O, Bidnenko S. Experience of antibacterial treatment of bone and joint sepsis of young children. Ann Intensive Care. 2016;6.

64. Kim HS, Whang DH, Lee KM. Comparison of multiplex real-time PCR with blood culture for identification of bloodstream pathogens. In Vivo. 2011;25(3):537.

65. Kitagawa Y, Ueda M, Ando N, Endo M, Ishibiki K, Kobayashi Y, et al. Rapid diagnosis of methicillin-resistant *Staphylococcus aureus* bacteremia by nested polymerase chain reaction. Ann Surg. 1996;224(5):665-71.

66. Kitagawa Y, Ueda M, Ando N, Ishibiki K, Kitajima M, Kabayashi Y, et al. [Rapid detection of mecA gene by nested PCR for diagnosis of methicillin resistance in *Staphylococcus aureus*]. Nihon Geka Gakkai Zasshi. 1992;93(9):914-7.

67. Knabl L, Mutschlechner W, Orth-Höller D. Evaluation of a multiplex OnSpot Primer-Extension PCR assay in the diagnosis of sepsis. J Microbiol Methods. 2016;120:91-3.

68. Korber F, Zeller I, Grünstäudl M, Willinger B, Apfalter P, Hirschl AM, et al. SeptiFast versus blood culture in clinical routine – A report on 3 years experience. Wien Klin Wochenschr. 2017;129(11-12):427-34.

69. Krulova B, Nemcova E, Zaloudikova B, Nemec P, Freiberger T. Comparison of commercial DNA extraction kits for the detection of bacterial genomic DNA from whole-blood samples using a broad-range PCR. Crit Care. 2009;13:S5.

70. Lehmann LE, Alvarez J, Hunfeld KP, Goglio A, Kost GJ, Louie RF, et al. Potential clinical utility of polymerase chain reaction in microbiological testing for sepsis. Crit Care Med. 2009;37(12):3085-90.

71. Lehmann LE, Hunfeld KP, Emrich T, Haberhausen G, Wissing H, Hoeft A, et al. A multiplex real-time PCR assay for rapid detection and differentiation of 25 bacterial and fungal pathogens from whole blood samples. Med Microbiol Immunol. 2008;197(3):313-24.

72. Leikeim RSM, Kesselmeier M, Löffler B, Rödel J, Höring S. Diagnostic accuracy and clinical impact of loop-mediated isothermal amplification for rapid detection of *Staphylococcus aureus* bacteremia: a retrospective observational study. Eur J Clin Microbiol Infect Dis. 2020;39(4):679-88.

73. Levi K, Towner KJ. Detection of methicillin-resistant *Staphylococcus aureus* (MRSA) in blood with the EVIGENE MRSA detection kit. J Clin Microbiol. 2003;41(8):3890-2.

74. Ley BE, Linton CJ, Bennett DM, Jalal H, Foot AB, Millar MR. Detection of bacteraemia in patients with fever and neutropenia using 16S rRNA gene amplification by polymerase chain reaction. Eur J Clin Microbiol Infect Dis. 1998;17(4):247-53.

75. Liberto MC, Puccio R, Matera G, Lamberti AG, Quirino A, Barreca GS, et al. Applications of LightCycler Staphylococcus M-GRADE assay to detect *Staphylococcus aureus* and coagulase-negative staphylococci in clinical blood samples and in blood culture bottles. Infez Med. 2006;14(2):71-6.

76. Liu CF, Shi XP, Chen Y, Jin Y, Zhang B. Rapid diagnosis of sepsis with TaqMan-Based multiplex real-time PCR. J Clin Lab Anal. 2018;32(2):e22256.

77. Long Y, Zhang Y, Gong Y, Sun R, Su L, Lin X, et al. Diagnosis of sepsis with cell-free DNA by next-generation sequencing technology in ICU patients. Arch Med Res. 2016;47(5):365-71.

78. Lucignano B, Ranno S, Liesenfeld O, Pizzorno B, Putignani L, Bernaschi P, et al. Multiplex PCR allows rapid and accurate diagnosis of bloodstream infections in newborns and children with suspected sepsis. J Clin Microbiol. 2011;49(6):2252-8.

79. Mahanta B, Das AK, Borthakur AK, Borah AK, Begum T, Anirvan P. Community acquired spontaneous bacterial peritonitis in cirrhotic patients and molecular concordance with bacteremia. Hepatol Int. 2018;12(2):S569.

80. Makhoul IR, Smolkin T, Sujov P, Kassis I, Tamir A, Shalginov R, et al. PCR-based diagnosis of neonatal staphylococcal bacteremias. J Clin Microbiol. 2005;43(9):4823-5.

81. Makhoul IR, Yacoub A, Smolkin T, Sujov P, Kassis I, Sprecher H. Values of C‐reactive protein, procalcitonin, and *Staphylococcus*‐specific PCR in neonatal late‐onset sepsis. Acta Paediatr. 2006;95(10):1218-23.

82. Mancilla J. Molecular detection of microorganism for the diagnosis of neonatal sepsis. J Perinat Med. 2013:41.

83. Mason WJ, Blevins JS, Beenken K, Wibowo N, Ojha N, Smeltzer MS. Multiplex PCR protocol for the diagnosis of staphylococcal infection. J Clin Microbiol. 2001;39(9):3332-8.

84. McHugh MP, Parcell BJ, MacKenzie FM, Templeton KE. Rapid molecular testing for *Staphylococcus aureus* bacteraemia improves clinical management. J Med Microbiol. 2020;69(4):552-7.

85. Mehta MS, McClure JT, Mangold K, Peterson LR. Performance of 3 real-time PCR assays for direct detection of *Staphylococcus aureus* and MRSA from clinical samples. Diagn Microbiol Infect Dis. 2015;83(3):211-5.

86. Metwally L, Gomaa N, Hassan R. Detection of methicillin-resistant *Staphylococcus aureus* directly by loop-mediated isothermal amplification and direct cefoxitin disk diffusion tests. East Mediterr Health J. 2014;20(4):273-9.

87. Midan DA, Abo El Fotoh WMM, El Shalakany AH. The potential role of incorporating real-time PCR and DNA sequencing for amplification and detection of 16S rRNA gene signatures in neonatal sepsis. J Matern Fetal Neonatal Med. 2017;30(12):1476-83.

88. Misawa Y, Yoshida A, Saito R, Yoshida H, Okuzumi K, Ito N, et al. Application of loop-mediated isothermal amplification technique to rapid and direct detection of methicillin-resistant *Staphylococcus aureus* (MRSA) in blood cultures. J Infect Chemother. 2007;13(3):134-40.

89. Mohamadi P, Kalantar E, Bahmani N, Fatemi A, Naseri N, Ghotbi N, et al. Neonatal bacteriemia isolates and their antibiotic resistance pattern in neonatal insensitive care unit (NICU) at Beasat hospital, Sanandaj, Iran. Acta Medica Iranica. 2014;52(5):337-40.

90. Mohammed HQ, Shehab AT. Molecular study of bacterial infection from hemodialysis patients in wasit centre, Iraq. Int J Res Pharm Sci. 2019;10(1):489-99.

91. Moore CC, Jacob ST, Banura P, Zhang J, Stroup S, Boulware DR, et al. Etiology of sepsis in Uganda using a quantitative polymerase chain reaction-based TaqMan array card. Clin Infect Dis. 2019;68(2):266-72.

92. Moore MS, McCarroll MG, McCann CD, May L, Younes N, Jordan JA. Direct screening of blood by PCR and pyrosequencing for a 16S rRNA gene target from emergency department and intensive care unit patients being evaluated for bloodstream infection. J Clin Microbiol. 2016;54(1):99-105.

93. Loonen AJM, Hansen WLJ, Jansz A, Kreeftenberg H, Bruggeman CA, Wolffs PFG, et al. Faster differentiation of *Staphylococcus aureus* versus coagulase-negative *Staphylococci* from blood culture material: A comparison of different bacterial DNA isolation methods. Crit Care. 2009;13:S4.

94. Nikolaras GP, Papaparaskevas J, Samarkos M, Tzouvelekis LS, Psychogiou M, Pavlopoulou I, et al. Changes in the rates and population structure of methicillin-resistant *Staphylococcus aureus* (MRSA) from bloodstream infections: A single-centre experience (2000-2015). J Glob Antimicrob Resist. 2019;17:117-22.

95. Notario R, Lejona S, Méndez E, All L, Lascialandare S, Borda N. Isolation of community-acquired meticillin-resistant *Staphylococcus aureus* (ca-mrsa) in Santa Fe province, Argentina. Revista Medica de Rosario. 2007;73(2):82-5.

96. Obara H, Aikawa N, Hasegawa N, Hori S, Ikeda Y, Kobayashi Y, et al. The role of a real-time PCR technology for rapid detection and identification of bacterial and fungal pathogens in whole-blood samples. J Infect Chemother. 2011;17(3):327-33.

97. Oeser C, Pond M, Butcher P, Bedford Russell A, Henneke P, Laing K, et al. PCR for the detection of pathogens in neonatal early onset sepsis. PLoS One. 2020;15(1):e0226817.

98. Ohlin A, Bäckman A, Björkqvist M, Mölling P, Jurstrand M, Schollin J. Real-time PCR of the 16S-rRNA gene in the diagnosis of neonatal bacteraemia. Acta Paediatr. 2008;97(10):1376-80.

99. Orszag P, Disqué C, Keim S, Lorenz MG, Wiesner O, Hadem J, et al. Monitoring of patients supported by extracorporeal membrane oxygenation for systemic infections by broad-range rRNA gene PCR amplification and sequence analysis. J Clin Microbiol. 2014;52(1):307-11.

100. Orszag P, Disqué C, Keim S, Lorenz MG, Haverich A, Kühn C. Broad-range pcr monitoring of bacteremia in patients supported by extracorporeal membrane oxygenation. Int J Med Microbiol. 2013;303:42.

101. Palomares JC, Puche B, Martos A, Lucena F, Marín M, Martín-Mazuelos E. Rapid molecular diagnosis of severe sepsis in patients with SIRS. Clin Microbiol Infect. 2009;15:S529-S30.

102. Papaparaskevas J, Brisse S, Verhoef J, Legakis NJ. Direct PCR amplification and sequencing of the 16S rRNA gene as a method for detection of bacteria in the blood. Acta Microbiol Hell. 2004;49(3):196-204.

103. Pasqualini L, Mencacci A, Leli C, Montagna P, Cardaccia A, Cenci E, et al. Diagnostic performance of a multiple real-time PCR assay in patients with suspected sepsis hospitalized in an internal medicine ward. J Clin Microbiol. 2012;50(4):1285-8.

104. Patel SS, Ghoshal U, Sahu C. Isolation of bacteria from blood samples of patients of apex trauma center with special reference to methicillin resistant *Staphylococcus aureus*. Indian J Pathol Microbiol. 2019;62(5):S81.

105. Pavone V, Lobreglio G, Pasanisi G, Maggio G, Cosi MC, Zecca C, et al. Molecular diagnosis approach of blood stream Infections of the neutropenic patient with haematological malignances. Haematologica. 2009;94:413.

106. Pechorsky A, Nitzan Y, Lazarovitch T. Identification of pathogenic bacteria in blood cultures: comparison between conventional and PCR methods. J Microbiol Methods. 2009;78(3):325-30.

107. Peters RP, van Agtmael MA, Gierveld S, Danner SA, Groeneveld AB, Vandenbroucke-Grauls CM, et al. Quantitative detection of *Staphylococcus aureus* and *Enterococcus faecalis* DNA in blood to diagnose bacteremia in patients in the intensive care unit. J Clin Microbiol. 2007;45(11):3641-6.

108. Pingle MR, Granger K, Feinberg P, Shatsky R, Sterling B, Rundell M, et al. Multiplexed identification of blood-borne bacterial pathogens by use of a novel 16S rRNA gene PCR-ligase detection reaction-capillary electrophoresis assay. J Clin Microbiol. 2007;45(6):1927-35.

109. Pomorska-Wesołowska M, Chmielarczyk A, Chlebowicz M, Ziółkowski G, Szczypta A, Natkaniec J, et al. Virulence and antimicrobial resistance of *Staphylococcus aureus* isolated from bloodstream infections and pneumonia in Southern Poland. J Glob Antimicrob Resist. 2017;11:100-4.

110. Punjabi NH, Taylor WRJ, Murphy GS, Purwaningsih S, Picarima H, Sisson J, et al. Etiology of acute, non-malaria, febrile illnesses in Jayapura, Northeastern Papua, Indonesia. Am J Trop Med Hyg. 2012;86(1):46-51.

111. Reier-Nilsen T, Farstad T, Nakstad B, Lauvrak V, Steinbakk M. Comparison of broad range 16S rDNA PCR and conventional blood culture for diagnosis of sepsis in the newborn: a case control study. BMC Pediatr. 2009;9:5.

112. Reuter CH, Palac HL, Kociolek LK, Zheng XT, Chao YY, Patel RM, et al. Ideal and actual impact of rapid diagnostic testing and antibiotic stewardship on antibiotic prescribing and clinical outcomes in children with positive blood cultures. Pediatr Infect Dis J. 2019;38(2):131-7.

113. Rogina P, Skvarc M, Stubljar D, Kofol R, Kaasch A. Diagnostic utility of broad range bacterial 16S rRNA gene PCR with degradation of human and free bacterial DNA in bloodstream infection is more sensitive than an in-house developed PCR without degradation of human and free bacterial DNA. Mediators Inflamm. 2014;2014:108592.

114. Rohit A, Maiti B, Shenoy S, Karunasagar I. Polymerase chain reaction-restriction fragment length polymorphism (PCR-RFLP) for rapid diagnosis of neonatal sepsis. Indian J Med Res. 2016;143(1):72-8.

115. Rozemeijer W, Peters RP, Kluytmans J, Savelkoul P, Schade RP. Kinetics of bacterial DNA in blood during endovascular infections with *Staphylococcus aureus*. Clin Microbiol Infect. 2010;16:S6.

116. Santolaya ME, Farfán MJ, De La Maza V, Cociña M, Santelices F, Alvarez AM, et al. Diagnosis of bacteremia in febrile neutropenic episodes in children with cancer: microbiologic and molecular approach. Pediatr Infect Dis J. 2011;30(11):957-61.

117. Sauget M, Bouiller K, Richard M, Chagrot J, Cholley P, Hocquet D, et al. Increasing incidence of bloodstream infections due to *Staphylococcus aureus* clonal complex 398 in a French hospital between 2010 and 2017. Eur J Clin Microbiol Infect Dis. 2019;38(11):2127-32.

118. Scanvic A, Courdavault L, Sollet JP, Le Turdu F. [Interest of real-time PCR Xpert MRSA/SA on GeneXpert(®) DX System in the investigation of staphylococcal bacteremia]. Pathol Biol (Paris). 2011;59(2):67-72.

119. Schaub N, Boldanova T, Noveanu M, Arenja N, Hermann H, Twerenbold R, et al. Incremental value of multiplex real-time PCR for the early diagnosis of sepsis in the emergency department. Swiss Med Wkly. 2014;144.

120. Seputiene V, Vilkoicaite A, Armalyte J, Pavilonis A, Suziedeliene E. Detection of methicillin-resistant *Staphylococcus aureus* using double duplex real-time PCR and dye syto 9. Folia Microbiol. 2010;55(5):502-7.

121. Shachor-Meyouhas Y, Sprecher H, Moscoviz D, Zaidman I, Haimi M, Kassis I. Molecular-based diagnosis of bacteremia in the setting of fever with or without neutropenia in pediatric hematology-oncology patients. J Pediatr Hematol Oncol. 2013;35(7):500-3.

122. Shin KS, Song HG, Kim H, Yoon S, Hong SB, Koo SH, et al. Direct detection of methicillin-resistant *Staphylococcus aureus* from blood cultures using an immunochromatographic immunoassay-based MRSA rapid kit for the detection of penicillin-binding protein 2a. Diagn Microbiol Infect Dis. 2010;67(3):301-3.

123. Silva-Junior WP, Martins AS, Xavier PC, Appel KL, Oliveira Junior SA, Palhares DB. Etiological profile of early neonatal bacterial sepsis by multiplex qPCR. J Infect Dev Ctries. 2016;10(12):1318-24.

124. Spencer DH, Sellenriek P, Burnham CA. Validation and implementation of the GeneXpert MRSA/SA blood culture assay in a pediatric setting. Am J Clin Pathol. 2011;136(5):690-4.

125. Spreafico M, Foglieni B, Raffaele L, Guarnori I, Berzuini A, Brigante G, et al. Applicability of a commercial real-time PCR assay, born for sepsis-causing microorganisms identification into the blood, to screening and detection of bacterial contamination in platelet concentrates. Vox Sanguinis. 2011;101:223.

126. Strålin K, Rothman RE, Özenci V, Barkataki K, Brealey D, Dhiman N, et al. Performance of PCR/Electrospray Ionization-Mass Spectrometry on whole blood for detection of bloodstream microorganisms in patients with suspected sepsis. J Clin Microbiol. 2020;58(9).

127. Stranieri I, Kanunfre KA, Rodrigues JC, Yamamoto L, Nadaf MIV, Palmeira P, et al. Usefulness of a 16S rDNA real-time PCR to monitor neonatal sepsis and to assist in medical decision to discontinue antibiotics. J Matern Fetal Neonatal Med. 2016;29(13):2141-4.

128. Straub J, Paula H, Mayr M, Kasper D, Assadian O, Berger A, et al. Diagnostic accuracy of the ROCHE Septifast PCR system for the rapid detection of blood pathogens in neonatal sepsis - A prospective clinical trial. PLoS One. 2017;12(11):e0187688.

129. Swathirajan C, Rameshkumar M, Solomon S, Pradeep A, Chithra D, Balakrishnan R, et al. Bacterial etiology and antibiotic resistance profile of bloodstream infections in human immunodeficiency virus patients from Southern India. J Res Med Sci. 2019;24(1):82.

130. Tat Trung N, Van Tong H, Lien TT, Van Son T, Thanh Huyen TT, Quyen DT, et al. Clinical utility of an optimised multiplex real-time PCR assay for the identification of pathogens causing sepsis in Vietnamese patients. Int J Infect Dis. 2018;67:122-8.

131. Tong MQ, Shang SQ, Wu YD, Zhao ZY. [Rapid diagnosis of neonatal sepsis by 16SrRNA genes PCR amplification and genechip hybridization]. Zhonghua Er Ke Za Zhi. 2004;42(9):663-7.

132. Tröger B, Härtel C, Buer J, Dördelmann M, Felderhoff-Müser U, Höhn T, et al. Clinical relevance of pathogens detected by multiplex PCR in blood of very-low-birth weight infants with suspected sepsis - multicentre study of the German neonatal network. PLoS One. 2016;11(7):e0159821.

133. Trung NT, Song LH. A simple pre-analytical tool to enrich bacterial DNA: Implications for blood sepsis diagnosis application. New Biotechnol. 2014;31:S108.

134. Tsalik EL, Jones D, Nicholson B, Caram LB, Liesenfeld O, Fowler VG, et al. Detection of bacterial and fungal pathogens associated with sepsis in patients presenting to the emergency room. Intensive Care Med. 2009;35:S196.

135. Tumuhamye J, Sommerfelt H, Bwanga F, Ndeezi G, Mukunya D, Napyo A, et al. Neonatal sepsis at Mulago national referral hospital in Uganda: Etiology, antimicrobial resistance, associated factors and case fatality risk. PLoS One. 2020;15(8):e0237085.

136. Turner RB, Lalikian K, Fry M, Schwartz J, Chan D, Won R. Impact of rapid identification of *Staphylococcus aureus* bloodstream infection without antimicrobial stewardship intervention on antibiotic optimization and clinical outcomes. Diagn Microbiol Infect Dis. 2017;89(2):125-30.

137. Ullrich E, Heidinger P, Soh J, Villanova L, Grabuschnig S, Bachler T, et al. Evaluation of host-based molecular markers for the early detection of human sepsis. J Biotechnol. 2020;310:80-8.

138. van de Groep K, Bos MP, Savelkoul PHM, Rubenjan A, Gazenbeek C, Melchers WJG, et al. Development and first evaluation of a novel multiplex real-time PCR on whole blood samples for rapid pathogen identification in critically ill patients with sepsis. Eur J Clin Microbiol Infect Dis. 2018;37(7):1333-44.

139. van den Brand M, Peters RPH, Catsburg A, Rubenjan A, Broeke FJ, van den Dungen FAM, et al. Development of a multiplex real-time PCR assay for the rapid diagnosis of neonatal late onset sepsis. J Microbiol Methods. 2014;106:8-15.

140. van den Brand M, van den Dungen FAM, Bos MP, van Weissenbruch MM, van Furth AM, de Lange A, et al. Evaluation of a real-time PCR assay for detection and quantification of bacterial DNA directly in blood of preterm neonates with suspected late-onset sepsis. Crit Care. 2018;22(1):105.

141. Vincent JL, Brealey D, Libert N, Abidi NE, O'Dwyer M, Zacharowski K, et al. Rapid diagnosis of infection in the critically ill, a multicenter study of molecular detection in bloodstream infections, pneumonia, and sterile site infections. Crit Care Med. 2015;43(11):2283-91.

142. Wallet F, Nseir S, Baumann L, Herwegh S, Sendid B, Boulo M, et al. Preliminary clinical study using a multiplex real-time PCR test for the detection of bacterial and fungal DNA directly in blood. Clin Microbiol Infect. 2010;16(6):774-9.

143. Wang LJ, Dong F, Qian SY, Yao KH, Song WQ. Clinical and Molecular epidemiology of invasive *Staphylococcus aureus* infections in Chinese children: A single-center experience. Chin Med J. 2017;130(23):2889-90.

144. Wang H. Early detection of antimicrobial-resistance pathogens using new methods. Int J Antimicrob Agents. 2015;45:S26.

145. Wellinghausen N, Siegel D, Gebert S, Winter J. Rapid detection of *Staphylococcus aureus* bacteremia and methicillin resistance by real-time PCR in whole blood samples. Eur J Clin Microbiol Infect Dis. 2009;28(8):1001-5.

146. Willey BM, Leung O, Rahman P, Trimi X, Grohn DN, Dunna S, et al. Comprehensive evaluation of cepheid's Xpert nasal complete G3 PCR (XNC) on their GeneXpert for distinguishing methicilin-resistant (MR) from methicillin-susceptible (MS) *Staphylococcus aureus* (SA) and coagulase-negative *Staphylococci* (CNS) from isolates. Can J Infect Dis Med Microbiol. 2015;26(2):e19-e20.

147. Wolk DM, Struelens MJ, Pancholi P, Davis T, Della-Latta P, Fuller D, et al. Rapid detection of *Staphylococcus aureus* and methicillin-resistant *S. aureus* (MRSA) in wound specimens and blood cultures: multicenter preclinical evaluation of the Cepheid Xpert MRSA/SA skin and soft tissue and blood culture assays. J Clin Microbiol. 2009;47(3):823-6.

148. Wu Q, Li Y, Hu H, Wang M, Wu Z, Xu W. Rapid identification of *Staphylococcus aureus*: FISH versus PCR methods. Lab Med. 2012;43(6):276-80.

149. Wu YD, Chen LH, Wu XJ, Shang SQ, Lou JT, Du LZ, et al. Gram stain-specific-probe-based real-time PCR for diagnosis and discrimination of bacterial neonatal sepsis. J Clin Microbiol. 2008;46(8):2613-9.

150. Wu YD, Shang SQ, Li JP, Yang ZQ, Zheng ZB, Du LZ, et al. [A broad-range 16S rRNA gene real-time PCR assay for the diagnosis of neonatal septicemia]. Zhonghua Er Ke Za Zhi. 2007;45(6):446-9.

151. Xiao Y, Shen X, Zhao QF, Yao YH, Yang TC, Niu JJ. Evaluation of real-time PCR coupled with multiplex probe melting curve analysis for pathogen detection in patients with suspected bloodstream infections. Front Cell Infect Microbiol. 2019;9:00361.

152. Yanagihara K, Kitagawa Y, Tomonaga M, Tsukasaki K, Kohno S, Seki M, et al. Evaluation of pathogen detection from clinical samples by real-time polymerase chain reaction using a sepsis pathogen DNA detection kit. Crit Care. 2010;14(4):R159.

153. Yue SJ, Hunan Neonatal Medical Quality Control C, Neonatology group of perinatal medical committee of Hunan Medical A. Recommendations on the diagnosis and the use of antibiotics for early-onset sepsis in preterm infants: Consensus of the expert panel from Hunan Province. Chin J Contemp Pediatr. 2020;22(1):1-6.

154. Zboromyrska Y, De la Calle C, Soto M, Sampietro-Colom L, Soriano A, Alvarez-Martínez MJ, et al. Rapid diagnosis of staphylococcal catheter-related bacteraemia in direct blood samples by real-time PCR. PLoS One. 2016;11(8):e0161684.

155. Zhang YC. Pathogen diagnosis of children sepsis by LAMP technology. Asian Pac J Trop Med. 2013;6(3):242-5.

156. Ziegler I, Fagerstrom A, Strålin K, Molling P. Evaluation of a commercial multiplex PCR assay for detection of pathogen DNA in blood from patients with suspected sepsis. PLoS One. 2016;11(12):e0167883.
